# LncRNA HAS2-AS1 Promotes Glioblastoma Proliferation by Sponging miR-137

**DOI:** 10.3389/fonc.2021.634893

**Published:** 2021-05-20

**Authors:** Yalin Lu, Gaochao Guo, Rujun Hong, Xingjie Chen, Yan Sun, Fang Liu, Zhimeng Zhang, Xun Jin, Jun Dong, Kai Yu, Xuejun Yang, Yang Nan, Qiang Huang

**Affiliations:** ^1^ Department of Neurosurgery, Tianjin Medical University General Hospital, Tianjin, China; ^2^ Key Laboratory of Post-Trauma Neuro-Repair and Regeneration in Central Nervous System, Ministry of Education and Tianjin City, Tianjin, China; ^3^ Tianjin Key Laboratory of Injuries, Variations and Regeneration of Nervous System, Tianjin, China; ^4^ Department of Neurosurgery, Henan Provincial People’s Hospital, People’s Hospital of Zhengzhou University, Zhengzhou, China; ^5^ Department of Psychiatry and Imaging-Genetics and Co-morbidity (PNGC Lab), Tianjin Anding Hospital, Tianjin Mental Health Center, Mental Health Teaching Hospital, Tianjin Medical University, Tianjin, China; ^6^ Department of Neurosurgery, Ningbo Hospital of Zhejiang University, Ningbo, China; ^7^ Tianjin Medical University Cancer Institute and Hospital, Tianjin, China; ^8^ National Clinical Research Center for Cancer, Tianjin Medical University Cancer Institute and Hospital, Tianjin, China; ^9^ Key Laboratory of Cancer Prevention and Therapy, Tianjin Medical University Cancer Institute and Hospital, Tianjin, China; ^10^ Tianjin’s Clinical Research Center for Cancer, Tianjin, China; ^11^ Department of Neurosurgery, The Second Affiliated Hospital of Soochow University, Soochow, China; ^12^ Department of Neurosurgery, Tianjin Medical University General Hospital Airport Site, Tianjin, China

**Keywords:** glioblastoma, lncRNA, ceRNA, miR-137, LSD1

## Abstract

GBM (Glioblastoma multiform) is the most malignant tumor type of the central nervous system and has poor diagnostic and clinical outcomes. LncRNAs (Long non-coding RNAs) have been reported to participate in multiple biological and pathological processes, but their underlying mechanism remains poorly understood. Here, we aimed to explore the role of the lncRNA HAS2-AS1 (HAS2 antisense RNA 1) in GBM. GSE103227 was analyzed, and qRT-PCR was performed to measure the expression of HAS2-AS1 in GBM. FISH (Fluorescence *in situ* hybridization) was performed to verify the localization of HAS2-AS1. The interaction between HAS2-AS1 and miR-137 (microRNA-137) was predicted by LncBook and miRcode followed by dual‐luciferase reporter assays, and the relationships among HAS2-AS1, miR-137 and LSD1 (lysine-specific demethylase 1) were assessed by WB (western blot) and qRT-PCR. Colony formation and CCK-8 (cell counting kit-8) assays were performed as functional tests. In vivo, nude mice were used to confirm the function of HAS2-AS1. HAS2-AS1 expression was upregulated in GBM cell lines, and HAS2-AS1 was localized mainly in the cytoplasm. *In vitro*, high HAS2-AS1 expression promoted proliferation, and knockdown of HAS2-AS1 significantly inhibited proliferation. Furthermore, HAS2-AS1 functioned as a ceRNA (competing endogenous RNA) of miR-137, leading to the disinhibition of its downstream target LSD1. The miR-137 level was downregulated by HAS2-AS1 overexpression and upregulated by HAS2-AS1 knockdown. In a subsequent study, LSD1 expression was negatively regulated by miR-137, while miR-137 reversed the LSD1 expression levels caused by HAS2-AS1. These results were further supported by the nude mouse tumorigenesis experiment; compared with xenografts with high HAS2-AS1 expression, the group with low levels of HAS2-AS1 exhibited suppressed proliferation and better survival. We conclude that lncRNA HAS2-AS1 promotes proliferation by functioning as a miR‐137 decoy to increase LSD1 levels and thus might be a possible biomarker for GBM.

## Introduction

Glioblastoma multiform is the most common primary carcinoma in adults, and standard treatments include tumor excision, radiotherapy and chemotherapy; however, the clinical outcomes of patients remain poor. The median survival time of patients is only 14 months, with a 5.5% five-year survival rate (1, 2). Numerous obstacles, such as the blood-brain barrier (3, 4), high tumor invasiveness (5), tumor heterogeneity (6), and temozolomide resistance (7), impede the effectiveness of GBM treatment. Thus, understanding the underlying mechanisms is critical for developing new strategies to improve curative effects.

Given that tumorigenesis requires many genetic mutations (8), many genes have been explored, such as IDH (isocitrate dehydrogenase), 1p/19q, and MGMT (O^6^-methylguanine-DNA methyltransferase) (9, 10), which mediate different pathological processes by distinct mechanisms and may be potential treatment targets. Recently, several studies found that immunodeficiency may participate in the processes of tumorigenesis in GBM (11, 12). During the past few decades, long noncoding RNAs, those longer than 200 base pairs in length without protein-coding capacity (13), have attracted the attention of researchers, as they are key regulators of diverse biological processes due to their function as ceRNAs by absorbing microRNAs (miRNAs) (14, 15). Aberrant lncRNA levels play vital roles in tumorigenic processes, such as tumor initiation and progression, chromosome instability, promotion of cell proliferation, resistance to apoptosis, and cancer metastasis (16–18). For example, Zhuoran Zhang et al. found that the lncRNA SBF2-AS1 enhances temozolomide resistance in GBM (19), and HAS2-AS1, which is located at the 8q24.13 locus, was reported to be an oncogene in oral squamous cell carcinoma (20). Another study demonstrated that HAS2-AS1 promotes the proliferation, invasion and growth of epithelial ovarian cancer (21). For GBM, only one study has demonstrated that HAS2-AS1 promotes tumor progression (22). We herein found that HAS2-AS1 promotes cell proliferation by sponging miR-137 to regulate LSD1 expression, which may provide a novel molecular mechanism of GBM.

## Materials and Methods

### Cell Culture

Six human GBM cell lines (LN18, U251, LNZ308, LN229, SNB19, U87MG) and human embryonic kidney 293T cells were purchased from American Type Culture Collection (Manassas, VA, USA). All GBM cells were cultured in high-glucose DMEM (Dulbecco’s modification of Eagle’s medium Dulbecco) (Corning, New York, USA) supplemented with 10% FBS (fetal bovine serum) (Thermo Fisher Scientific, Massachusetts, USA) at 37°C and 5% CO_2_.

### Plasmids, siRNA and Lentivirus

The pcDNA3.1-PGK-EGFP plasmid overexpressing HAS2-AS1, sequences of siRNA for HAS2-AS1, scrambled siRNAs, miR-137 mimics, miR-137 NC mimics, miR-137 inhibitor, and miR-137 NC inhibitor were in **Table S1** (Hanbio, Shanghai, China). The pSICHECK2.0 vector was used for dual-luciferase assays, and the sequences were as follows:

wild-type: 5’-···GTGTGACTTAGCAATATGTTGTTGCTGACCT···-3’ and
mutant: 5’-··· GTGTGACTTCGAACTCTGTTGTTGCTGACCT···-3’.

Lentivirus for knocking down HAS2-AS1 was purchased from Hanbio, and all experiments were performed according to the manufacturers’ protocols. After infection, cells were selected using a 5 µg/ml puromycin solution (Thermo Fisher Scientific, Massachusetts, USA).

### Cell Transfection and Culture

U87MG and U251 cells were seeded in 6- and 12-well plates and transfected 48 h later using Lipofectamine 3000 (Life Technologies Corporation, Gaithersburg, MD, USA) according to the manufacturer’s protocol.

### RNA Extraction and RT-qPCR

Total RNA was extracted from the cells by TRIzol (TaKaRA, China) according to the manufacturer’s protocol. cDNA was synthesized using the GoScript Reverse Transcription System (Promega, Wisconsin, USA) and 1 µg of RNA as described by the manufacturer. qPCR was performed using a SYBR Green PCR kit (Biomake, Houston, USA) according to the manufacturer’s instructions on an Applied Biosystems QuantStudio instrument (Thermo Fisher Scientific, Massachusetts, USA). Comparative quantification was performed using the 2DCt method with GAPDH/U6 as the endogenous control. The primer sequences were in **Table S2** (Tianyihuiyuan, China): The Catalog numbers of RT primers, forward and reverse primers for miR-137/U6wereshown in **Table S3** (Ribobio, Guangzhou, China).

### Western Blot Assay

RIPA buffer (Solarbio, R0010, China) containing protease inhibitors (Solarbio, A8260, China) was used to isolate proteins from U251 and U87MG cells, and the protein concentrations were determined by a BCA kit (Solarbio, PC0020, China). Then, denatured proteins were separated by SDS-PAGE (sodium dodecyl sulfate-polyacrylamide gel electrophoresis) and transferred onto PVDF membranes (Millipore, Billerica, USA). The membranes were blocked in TBST with 5% skim milk for 1 h and then incubated with appropriate anti-human antibodies at 4°C overnight; the specific protein-antibody complex was detected by horseradish peroxidase-conjugated goat anti-rabbit or rabbit anti-mouse IgG according to the manufacturer’s instructions. All antibodies were purchased from Cell Signaling Technology (Massachusetts, USA; catalog numbers: GAPDH: #5174S 1:1000 dilution; LSD1: #2139S 1:1000 dilution; Anti-mouse IgG: #7076S 1:2000 dilution; Anti-rabbit IgG: #7074S 1:2000 dilution). Finally, we detected protein signals with a Bio-Rad ChemiDoc XRS system (Bio-Rad, Hercules, CA). The GAPDH signal was used as the loading control.

### CCK-8 Assay

After transfection, U87MG and U251 cells were divided into 96-well plates at 3×10³ per well. At 0, 24, 48, 72, 96, 120, and 144 h, CCK-8 solution was added (10 µl, Beyotime, China), and the cells were then incubated for 2 h at 37°C and 5% CO_2_. Then, the OD (optical density) at 450 nm (OD450) was detected by a microplate reader (Biotek, Vermont, USA).

### Colony Formation Assay

Forty-eight hours after the transduction of U87MG and U251 cells, approximately 500 cells were cultured in each well of a 6-well plate for 2 weeks. Afterwards, cells were fixed in 4% phosphate-buffered paraformaldehyde (Solarbio, China) and then dyed with 0.1% crystal violet (Solarbio, China). ImageJ software (ImageJ, NIH) was used to determine the colony formation rate.

### Dual-Luciferase Reporter Assays

The luciferase reporter gene vector loaded with HAS2-AS1 WT/MUT was cotransfected with miR-137 mimics/inhibitor into 293T cells by Lipofectamine 3000 reagent (Thermo Fisher Scientific, Massachusetts, USA). Luciferase activity was detected using the Dual-Luciferase^®^ Reporter Assay System (Promega, Madison, Wisconsin, USA), and the experiment was repeated at least three times.

### Fluorescence In Situ Hybridization

All processes were performed in accordance with the manufacturer’s protocol (GenePharma, China). Slides were preheated in the oven at 60°C for 30 min to melt the paraffin, after which they were immersed in xylene and a graded ethanol series (100%, 95%, 90%, 80%, 70%) for 1 min each. Then, the samples were washed twice with PBS for 2 min each time, followed by digestion with proteinase K for 20 min at 37°C. Next, 2× buffer C was added to each slide three times for 1 min each, and the slides were immersed in a graded ethanol series (70%, 80%, 90%, 100%) for 2 min each and allowed to dry. Denaturation liquid was added for 8 min at 78°C, and dehydration was performed by an ethanol gradient. The HAS2-AS1 RNA probe was diluted to 2 μM. Each sample was covered with 10 μL of diluted probe and incubated in a humidified hybridization chamber at 37°C for 12 h. Slides were sequentially washed in wash solution (43°C) for 15 min, 2×buffer C for 10 min, and PBS for 1 min. Subsequently, DAPI was added for 20 min in the dark, and the slides were washed with PBS twice for 2 min each time. The slides were dried with an antiquenching agent and covered with a cover glass. Images were obtained using a microscope (Olympus, Japan). Three different random images were obtained for each sample at 400× magnification, and the relative density of the HAS2-AS1 signal was quantified by ImageJ software. The results were analyzed by paired t-test, and p < 0.05 was considered to be statistically significant.

### IHC (Immunohistochemistry)

Brain tumors were immersed in 4% formalin, embedded in paraffin, cut into slices, and dewaxed. After antigen retrieval, the slides were blocked with BSA (bovine serum albumin) for 30 min at 37°C and then incubated with primary antibodies against Ki-67 (Zsbio, ZA-0502, 1:100 dilution) at 4°C overnight. Afterwards, the slides were rewarmed at room temperature for 1 h and then incubated with a biotin-labeled secondary antibodies (Zsbio, SP-9001). The DAB (diaminobenzidine) kit (Solarbio, DA1015) was used for staining, and hematoxylin was used for counterstaining. Finally, the samples were dehydrated, and the images were acquired by microscopy (Olympus, Japan).

### Nude Mouse Tumorigenesis Experiment

A total of 10 six-week-old male nude mice were selected for xenograft assays. The mice were randomly divided into 2 groups with 5 mice in each group: control group and sh-HAS2-AS1 group. In brief, 5×10^6^ U87MG cells (pretreated with sh-HAS2-AS1 lentivirus or scrambled shRNA) were implanted into brains using a stereotactic instrument. Thereafter, the tumors were measured every 7 days by bioluminescence imaging. The processes were as follows: after anesthesia, the mice were intraperitoneally injected with D-luciferin, and the IVIS (In Vivo Imaging System) was used for imaging. All animal assays were performed in accordance with the Animal Welfare Act and approved by the Institutional Committee for Animal Research of Tianjin Medical University.

### Statistical Analysis

The results were analyzed using GraphPad software 6.0 (GraphPad Software, La Jolla, CA, USA) and were shown as the means ± SDs, the experiment was repeated three times independently. Student′s t-test was performed to compare differences in the two different groups with parametric variables. Overall survival was evaluated using the Kaplan-Meier method and log rank test. P <0.05 was considered statistically significant. Variance is similar between the groups that are being statistically compared.

## Results

### LncRNA HAS2-AS1 Is Highly Expressed in glioma and Negatively Correlated With Survival

**Figure 1:**
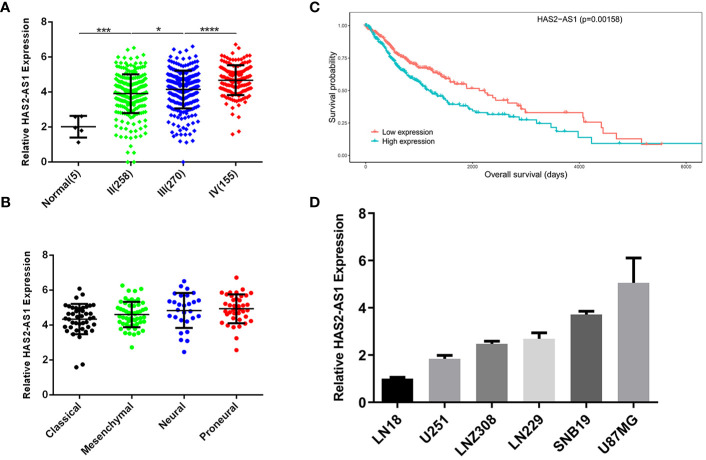
LncRNA HAS2-AS1 is highly expressed in glioma and negatively correlated with survival. **(A)** HAS2-AS1 expression in gliomas of different WHO grades from the GEO database. **(B)** Kaplan-Meier curves were generated between HAS2-AS1 and overall survival. The median HAS2-AS1 expression was used as a cutoff. **(C)** HAS2-AS1 expression in different GBM subtypes from the GEO database. **(D)** The levels of HAS2-AS1 in different GBM cell lines (LN18, U251, LNZ308, LN229, SNB19, U87MG) were analyzed by qRT-PCR. qRT-PCR was repeated three times independently. *P < 0.05, ***P < 0.001, ****P < 0.0001.

To identify the expression of HAS2-AS1 in glioma, we searched the GEO (Gene Expression Omnibus) database (https://www.ncbi.nlm.nih.gov/geo/), and GSE103227 attracted our attention. The level of HAS2-AS1 was positively correlated with the WHO grade of glioma (**Figure 1A**), which indicated that HAS2-AS1 may be involved in the tumorigenesis processes in GBM. We also explored the relationship between HAS2-AS1 expression and GBM classifications and found no differences among the subtypes (**Figure 1B**). Kaplan-Meier curves were used to confirm the correlation between HAS2-AS1 and overall survival, revealing that the level of HAS2-AS1 was negatively correlated with overall survival (**Figure 1C**). Next, we assessed the expression of HAS2-AS1 in different GBM cell lines (LN18, U251, LNZ308, LN229, SNB19, U87MG) by qRT-PCR (**Figure 1D**), and U87MG and U251 cells were selected for further experiments. These results showed that lncRNA HAS2-AS1 was overexpressed in GBM.

### HAS2-AS1 Promotes Proliferation *In Vitro* and *In Vivo*

**Figure 2:**
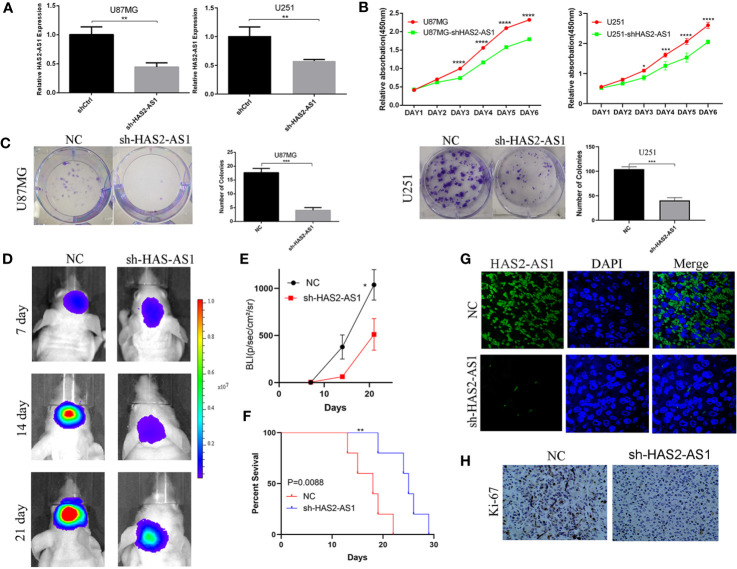
HAS2-AS1 promotes proliferation *in vitro*, and HAS2-AS1 knockdown inhibits proliferation *in vivo*. **(A)** HAS2-AS1 expression in U87MG and U251 cells transfected with sh-HAS2-AS1 or shCtrl lentivirus was measured by qRT-PCR. GAPDH was used as an internal control. **(B)** CCK-8 assays were conducted to assess the proliferative abilities of U251 and U87MG cells after HAS2-AS1 knockdown. **(C)** Colony formation assays were performed to determine the proliferation potential after HAS2-AS1 knockdown in U251 and U87MG cells. **(D)** Representative bioluminescence images of intracranial xenografts implanted with U87MG HAS2-AS1-knockdown or shCtrl cells and the BLI **(E)** on days 7, 14, and 21. **(F)** The survival curves of intracranial xenografts of nude mice injected with U87MG HAS2-AS1-knockdown or shCtrl cells. **(G)** FISH showed that HAS2-AS1 was mainly localized in the cytoplasm, and HAS2-AS1 expression in intracranial xenografts was significantly reduced after HAS2-AS1 knockdown. **(H)** IHC showed that Ki-67 expression in intracranial xenografts was reduced after HAS2-AS1 knockdown. The experiment was repeated three times independently. The data are measurements and are expressed as the mean ± standard deviation. *P < 0.05, **P < 0.01, ***P < 0.001, ****P < 0.0001.

To explore the biological functions of HAS2-AS1 during GBM proliferation, loss-of-function assays were performed in U87MG and U251 cells. After transfection, qRT-PCR was performed to evaluate the knockdown effects (**Figure 2A**), and CCK-8 assays demonstrated that knockdown of HAS2-AS1 inhibited the proliferation of U87MG and U251 cells (**Figure 2B**). Colony formation assays were also implemented, revealing that compared to the normal control group, the sh-HAS2-AS1 group exhibited significantly fewer colonies (**Figure 2C**). To further investigate the effect of HAS2-AS1, animal experiments were conducted, during which we injected HAS2-AS1-knockdown or control U87MG cells into nude mice (KD group: n=5, NC group: n=5). Every 7 days, tumor volumes were measured by an IVIS and were shown to be significantly decreased after HAS2-AS1 knockdown (**Figure 2D**). The BLI (bioluminescence intensity) experiment showed that HAS2-AS1 knockdown inhibited tumor proliferation and increased mouse survival (**Figure 2E**). The survival curves of nude mice were significantly better in the U87MG sh-HAS2-AS1 group (**Figure 2F**). In addition, FISH assays demonstrated that HAS2-AS1 was expressed at a lower level in the knockdown group than in the control group (**Figure 2G**), and IHC staining revealed lower ki-67 levels after knockdown of HAS2-AS1 than in the control group (**Figure 2H**). These results showed that HAS2-AS1 knockdown suppressed proliferation *in vitro* and *in vivo*.

### HAS2-AS1 Functions as a ceRNA for miR‐137

**Figure 3:**
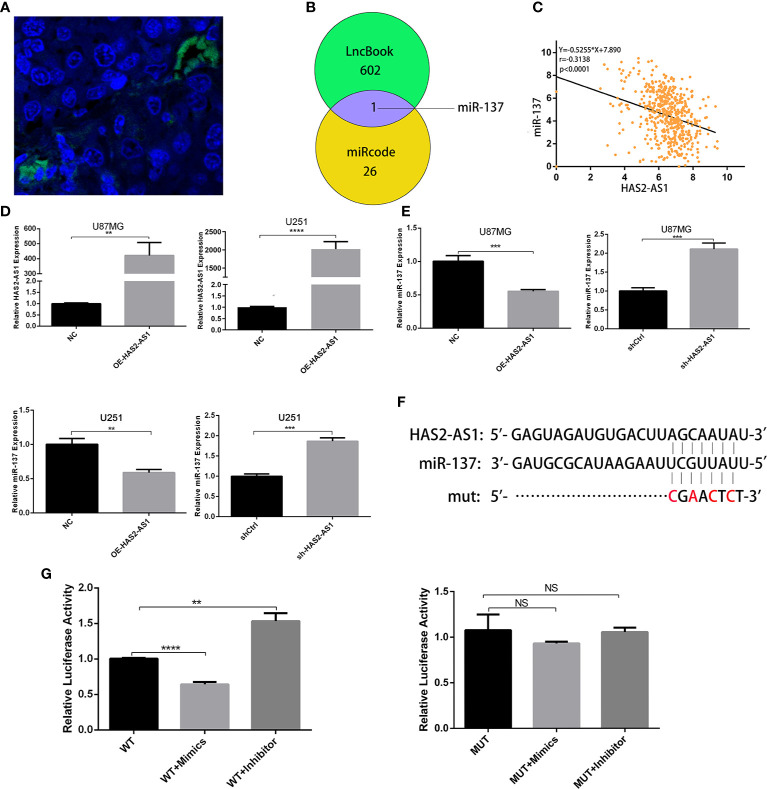
HAS2-AS1 functions as a competing endogenous RNA for miR‐137. **(A)** FISH showed that HAS2-AS1 was mainly localized in the cytoplasms of nude mouse xenografts. **(B)** The bioinformatics analysis of potential miRNAs predicted by LncBook and miRcode. **(C)** Association analysis of the relationship between HAS2-AS1 and miR-137 predicted by the TCGA. **(D)** HAS2-AS1 expression in U87MG and U251 cells transfected with an HAS2-AS1 overexpression plasmid or empty vector was measured by qRT-PCR. GAPDH was used as an internal control. **(E)** miR-137 expression was measured by qRT-PCR after transfection of U87MG and U251 cells overexpressing the HAS2-AS1 plasmid or sh-HAS2-AS1 lentivirus. **(F)** Schematic diagram of potential miR-137 binding sites at HAS2-AS1 (WT) and mutation (mut) sites. **(G)** The luciferase reporter plasmid containing wild-type/mutant HAS2-AS1 was cotransfected into 293T cells with miR-137 mimics/inhibitor. After 48 h, dual-luciferase reporter assays were performed. The experiment was repeated three times independently. The data are measurements and expressed as the mean ± standard deviation. **P < 0.01, ***P < 0.001, ****P < 0.0001, NS, no significance.

To further explore how HAS2-AS1 promotes GBM proliferation, we performed FISH to determine HAS2-AS1 localization, revealing that it mainly resided in the cytoplasm (**Figure 3A**). We hypothesized that HAS2-AS1 may function as a “sponge” of microRNA. According to bioinformatics analysis by LncBook (https://bigd.big.ac.cn/lncbook/index) and miRcode (http://mircode.org/index.php), miR-137 is the only candidate predicted to interact with HAS2-AS1 (**Figure 3B**). We then bioinformatically analyzed the correlation between HAS2-AS1 and miR-137, revealing a significant negative correlation (**Figure 3C**). Considering the biases of bioinformatics analysis, we performed qRT-PCR to verify the results. Similarly, the miR-137 level was downregulated when HAS2-AS1 was overexpressed, while miR-137 was upregulated when HAS2-AS1 was knocked down (**Figures 3D, E** **)**. Additionally, we mutated the potential binding sites of HAS2-AS1 and tested the construct in the luciferase assay (**Figure 3F**). The luciferase activities were significantly decreased when the miR-137 mimics were used, while they were increased after transduction of the miR-137 inhibitor into the wild-type group. Moreover, no differences were observed among the mutated groups (**Figure 3G**). In brief, HAS2-AS1 can sponge miR-137.

### LSD1 Is a Target Gene of miR-137 and Is Regulated by HAS2-AS1

**Figure 4:**
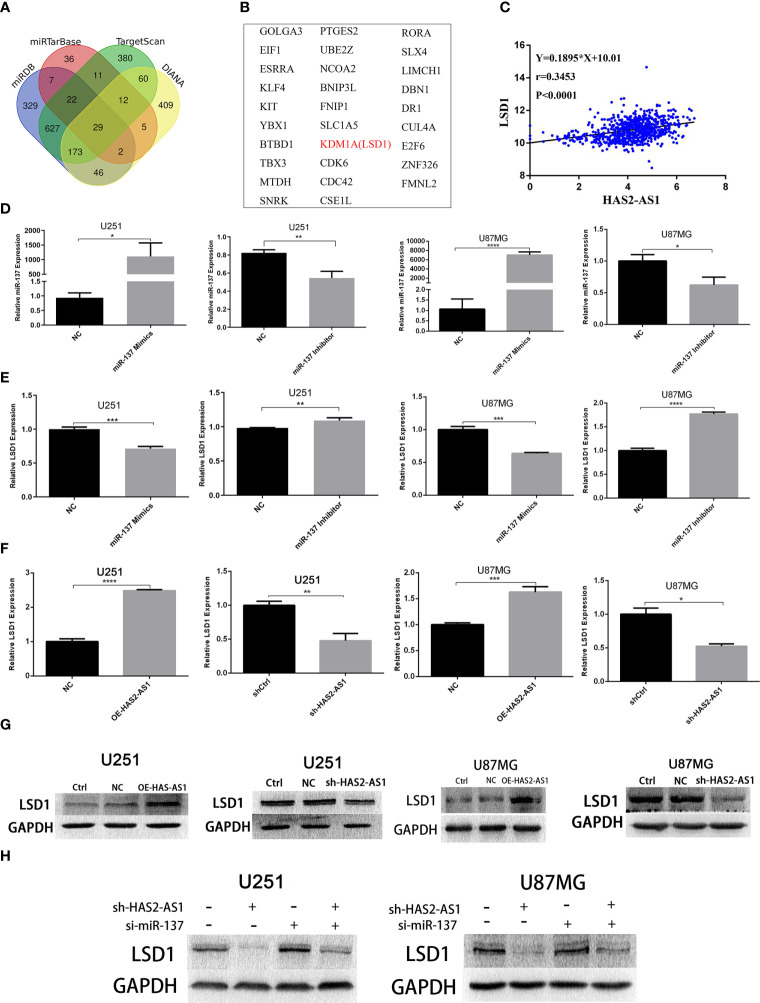
LSD1 is a target gene of miR-137 and is regulated by HAS2-AS1. **(A)** Bioinformatics analysis predicted the potential mRNAs shared between TargetScan, miRTarBase, miRDB and DIANA, 29 potential mRNAs **(B)** were identified. **(C)** The relationship between HAS2-AS1 and LSD1 was predicted by TCGA. **(D)** The miR-137 expression in U87MG and U251 cells after transduction of miR-137 mimics/inhibitor was validated by qRT-PCR. **(E)** The relative expression of LSD1 in U87MG and U251 cells after transduction of the miR-137 mimics/inhibitor as measured by qRT-PCR. **(F)** The relative expression of LSD1 in U87MG and U251 cells after overexpression/knockdown of HAS2-AS1 as determined by qRT-PCR. **(G)** The relative protein levels of LSD1 in U87MG and U251 cells after HAS2-AS1 overexpression/knockdown were confirmed by Western blot. GAPDH was used as a loading control. **(H)** The miR-137 inhibitor reversed the change in LSD1 expression caused by HAS2-AS1 silencing at the protein level. The experiment was repeated three times independently. The data are measurements and expressed as the mean ± standard deviation. *P < 0.05, **P < 0.01, ***P < 0.001, ****P < 0.0001.

To explore the targets of miR-137, we searched TargetScan (http://www.targetscan.org/vert_71/),miRTarBase (http://mirtarbase.cuhk.edu.cn/php/index.php),miRDB (http://mirdb.org/), and DIANA (http://carolina.imis.athena-innovation.gr/diana_tools/web/index.php?r=tarbasev8/index) and identified 29 candidates in total (**Figures 4A, B** **)**, among which LSD1 attracted our attention. First, we found thatLSD1 was negatively correlated with miR-137 (**Figure 4C**). Confirmation of the effects of the miR-137 mimics and inhibitor were confirmed by qRT-PCR (**Figure 4D**), and a remarkable negative correlation between miR-137 and LSD1 was observed (**Figure 4E**). miR-137 mimics suppressed the expression of LSD1 in U251 and U87MG cells, while the miR-137 inhibitor promoted LSD1 expression. Next, we performed qRT-PCR to investigate the relationship between HAS2-AS1 and LSD1. As shown in **Figure 4F**, LSD1 expression was upregulated when HAS2-AS1 was overexpressed but decreased when HAS2-AS1 was knocked down. Western blot analysis demonstrated the same trends in U251 and U87MG cells (**Figure 4G**), and these results showed that HAS2-AS1 can regulate LSD1. Furthermore, we also performed rescue assays by western blot (**Figure 4H**), revealing that the miR-137 inhibitor reversed LSD1 expression when HAS2-AS1 was knocked down in U251 and U87MG cells. These results demonstrated that LSD1 can be regulated by miR-137 and HAS2-AS1.

## Discussion

Increasing evidence supports that lncRNAs play important roles in tumorigenesis, growth, invasion, and metastasis (8). HAS2-AS1, located on chromosome 8, is the antisense RNA 1 of HAS2. HIF-1α was first reported to activate the transcription of HAS2-AS1 in oral squamous cell carcinoma under hypoxic conditions, and high levels of HAS2-AS1 were correlated with epithelial mesenchymal transition and tumor metastasis (20). Another study revealed that HAS2-AS1 promotes proliferation and invasion through the miR-466/RUNX2 axis in epithelial ovarian cancer (21). In glioma, HAS2-AS1 knockdown inhibits cell proliferation, migration and invasion *via* the PI3K/AKT signaling pathway (23). Another recent study showed that HAS2-AS1 sponges miR-608 to regulate PRPS1 and thus promote GBM progression (22). We herein determined that lncRNA HAS2-AS1 expression was upregulated in GBM and positively correlated with advanced stages and poor prognosis, leading to the hypothesis that it may participate in the tumorigenesis process. Then, we performed CCK-8 and colony formation assays, and the results confirmed our hypothesis, as knocking down HAS2-AS1 obviously suppressed GBM cell proliferation. Furthermore, those results were further supported by the nude mouse tumorigenesis experiment, which showed that compared with xenografts expressing high levels of HAS2-AS1, the group with low levels of HAS2-AS1 exhibited suppressed proliferation and better survival. Therefore, HAS2-AS1 has the potential to be a novel and valuable oncogene for GBM.

The mechanisms of lncRNAs have been well studied based on interactions with miRNAs, with one example being lncRNAs acting as “miRNA sponges”. Margaret et al. was the first to develop miRNA inhibitors that could inhibit targeting miRNAs *via* binding sites and termed them “miRNA sponges” (24), afterwards, Salmena et al. summarized miRNA interaction mechanisms and named them the “ceRNA network”, in which lncRNAs interact with miRNAs by binding sequences and reverse the inhibition of mRNAs caused by miRNAs (25). MiRNAs play critical roles in the posttranscriptional regulation of biological processes by mediating mRNA degradation or translational repression, and they bind the 3’- UTR (untranslated region) of target mRNAs (26, 27). The RISC (RNA-induced silencing complex) is necessary for this process (28). Scientific and technologic advancements, especially regarding gene sequencing and bioinformatics (29, 30), have led to the detection of numerous miRNAs in various diseases. For example, Liu et al. showed that lncNB1 promotes tumorigenesis by reacting with the ribosomal protein RPL35 in neuroblastoma (31). LncRNA MIR4435-2HG can sponge miR-1224-5p to regulate TGFBR2 expression, which mediates the proliferation and invasion of GBM (32). Ma et al. found that lncRNA GCASPC can interact with miR-17-3p, thus decreasing PC expression to influence the proliferation of gallbladder cancer (33). Liu et al. expounded LncRNA HNF1A-AS1 promotes progression partly by competitively binding to miR-661 to enhance the expression of cell division cycle 34 in gastric cancer (34). In chronic myelogenous leukemia, G. Silvestri et al. illustrated that MIR300 is crucial for induction and maintenance of LSC quiescence and impaired NK cell anticancer immunity, and lncRNA TUG1 selectively suppressed MIR300 proapoptotic activity (35). Huaying Dong et al. proved lncRNA TINCR can sponge miR-125b, thus releasing HER-2 and inducing trastuzumab resistance in breast cancer (36).

MiR-137 is enriched in the brain and plays critical roles in neural stem cell proliferation and differentiation during neural development (37). In malignant pleural mesothelioma, miR-137 and its downstream target YBX1 promote tumor invasion (38). In multiple myeloma, miR-137 is downregulated, thus inducing drug resistance and chromosomal instability by targeting AURKA (39). In pancreatic cancer, miR-137 inhibits tumorigenesis by targeting KLF12 (40). In triple-negative breast cancer, miR-137 inhibits cancer progression by targeting DEL-1 (41).

Herein, to investigate the mechanism underlying HAS2-AS1, we first performed FISH and found that it was mainly located in the cytoplasm, indicating that it may function as a miRNA sponge. Bioinformatic analyses showed that miR-137 was regulated by HAS2-AS1, and this result was supported by that of the dual-luciferase reporter assay. Additionally, miR-137 was downregulated when HAS2-AS1 was overexpressed as determined by qRT-PCR, whereas miR-137 was upregulated when HAS2-AS1 was knocked down. Thus, HAS2-AS1 could act as a sponge of miR-137, and miR-137 may function as an antioncogene for GBM.

**Figure 5:**
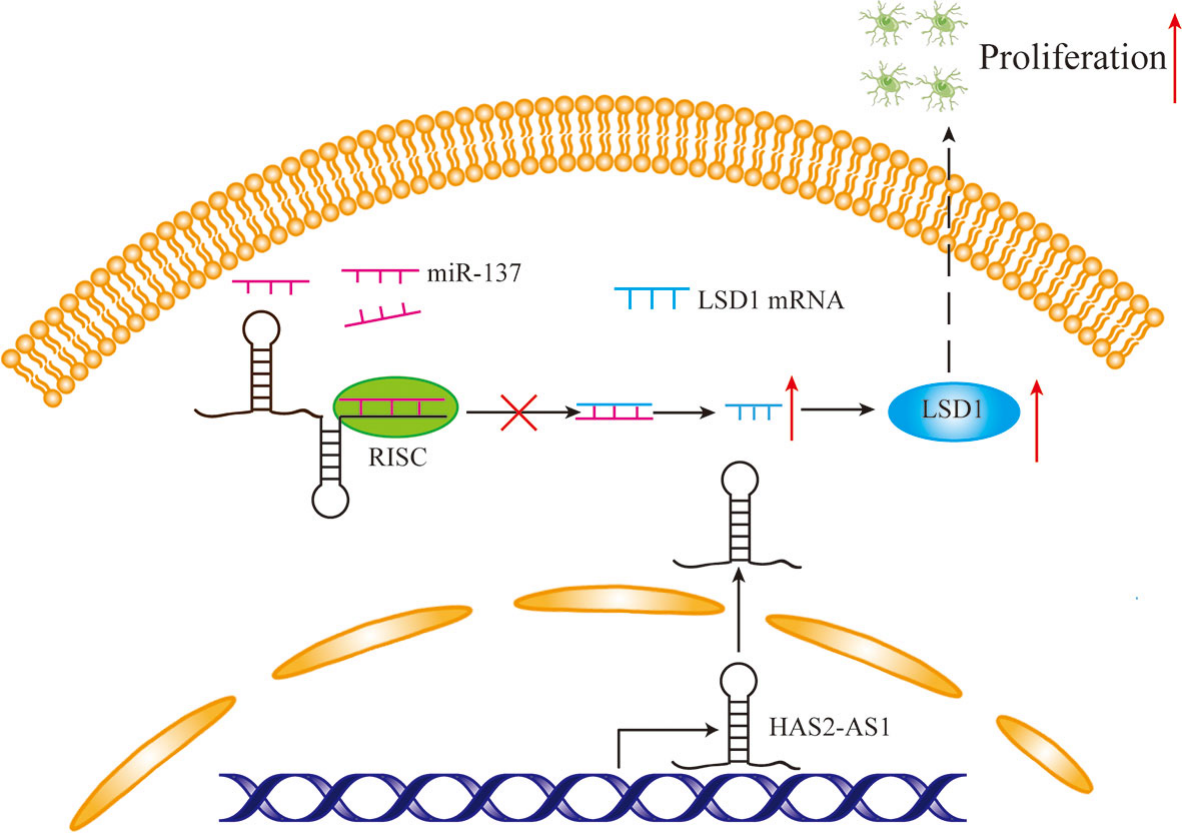
The mechanism diagram of HAS2-AS1, miR-137 and LSD1. Overexpressed HAS2-AS1 functions as a ceRNA by competitively binding to miR-137 to upregulate LSD1 expression, thereby promoting GBM proliferation.

In addition, LSD1, also called KDM1A, functions as a histone demethylase, specifically demethylating H3K4me1/2 and H3K9me1/2 (42). LSD1 also regulates certain non-histone substrates that play important roles in gene expression (43). Numerous studies have demonstrated that high levels of LSD1 participate in many diverse cancers, especially lung cancer (44) and acute myeloid leukemia (45, 46). Keri et al. showed that an LSD1 inhibitor could repress cell migration in medulloblastoma (47). Kei Yi et al. demonstrated that LSD1 promotes GBM cell tumorigenesis and metastasis (48). Furthermore, several studies have shown that LSD1 is a target of miR-137, and Francesc et al. determined that LSD1 is the mRNA target of miR-137 in colorectal cancer (49). Kristina et al. showed that miR-137 downregulated LSD1 to suppress tumor aggressiveness in neuroblastoma (50). Xin Zhang et al. obtained similar results in non-small cell lung cancer (51). In this study, we found that LSD1 was a candidate of miR-137 by bioinformatic analyses and negatively correlated with HAS2-AS1. We then performed qRT-PCR and WB, revealing that LSD1 was regulated by lncRNA HAS2-AS1, and this regulatory relationship was reversed by miR-137. Thus, the regulatory network of HAS2-AS1/miR-137/LSD1 could play an important role in GBM proliferation (**Figure 5**).

## Conclusions

In conclusion, we demonstrated that lncRNA HAS2-AS1 sponges miR-137 to increase the expression of LSD1, consequently promoting GBM proliferation. Our results reveal that this lncRNA might be an oncogene and provide a therapeutic target for GBM.

## Data Availability Statement

The original contributions presented in the study are included in the article/**Supplementary Material**. Further inquiries can be directed to the corresponding authors.

## Ethics Statement

The experimental design for this study was approved by the Ethics Committee and Experimental Animal Ethics Committee of Tianjin Medical University General Hospital, Tianjin Medical University.

## Author Contributions

QH and YN conceived and designed the experiments. YL, GG and RH performed the experiments. XC, YS, FL, ZZ, XJ, JD, KY and XY contributed reagents/materials/analysis tools. All authors contributed to the article and approved the submitted version.

## Funding

This work was supported by the National Natural Science Foundation of China (grant Nos. 81572490 and 81172405), Science and Technology fund of Tianjin Binhai New Area Health and Family Planning Commission (grant Nos. 2018BWKZ002 and 2018BWKZ003) and Tianjin Science and Technology Committee (grant No. 18JCZDJC98600).

## Conflict of Interest

The authors declare that the research was conducted in the absence of any commercial or financial relationships that could be construed as a potential conflict of interest.

## Supplementary Material

Supplementary Figure 1 **(A)** HAS2-AS1 expression in PTC, PC and TJ905 cells transfected with siRNA for HAS2-AS1 or scrambled siRNA was measured by qRT-PCR. GAPDH was used as an internal control. **(B)** CCK-8 assays were conducted to assess the proliferative abilities of PTC, PC and TJ905 cells after HAS2-AS1 knockdown. **(C)** Colony formation assays were performed to determine the proliferation potential after HAS2-AS1 knockdown in PTC, PC and TJ905 cells.

Supplementary Figure 2 **(A)** Relative fluorescence intensity of **Figure 2G**. **(B)** The percentage of Ki-67-positive cells of **Figure 2H**. **(C)** Ratio of the LSD1 expression level normalized to that in the control group after overexpression/knockdown of HAS2-AS1 as measured by WB in LNZ308 cells. **(D)** Ratio of the LSD1 expression level normalized to that in the control group after overexpression/knockdown of HAS2-AS1 as measured by WB in U87 cells. **(E)** The ratio of the LSD1 expression levels in different groups of LNZ308 and U87 cells normalized to that in the control group. The results showed that the miR-137 inhibitor reversed the change in LSD1 expression caused by HAS2-AS1 silencing at the protein level.
