# Supplementary figures and images for: LncRNA HAS2-AS1 Promotes Glioblastoma Proliferation by Sponging miR-137
Source: Front Oncol. 2021 May 20;11:634893. doi: 10.3389/fonc.2021.634893 (PMC8173206; doi:10.3389/fonc.2021.634893)

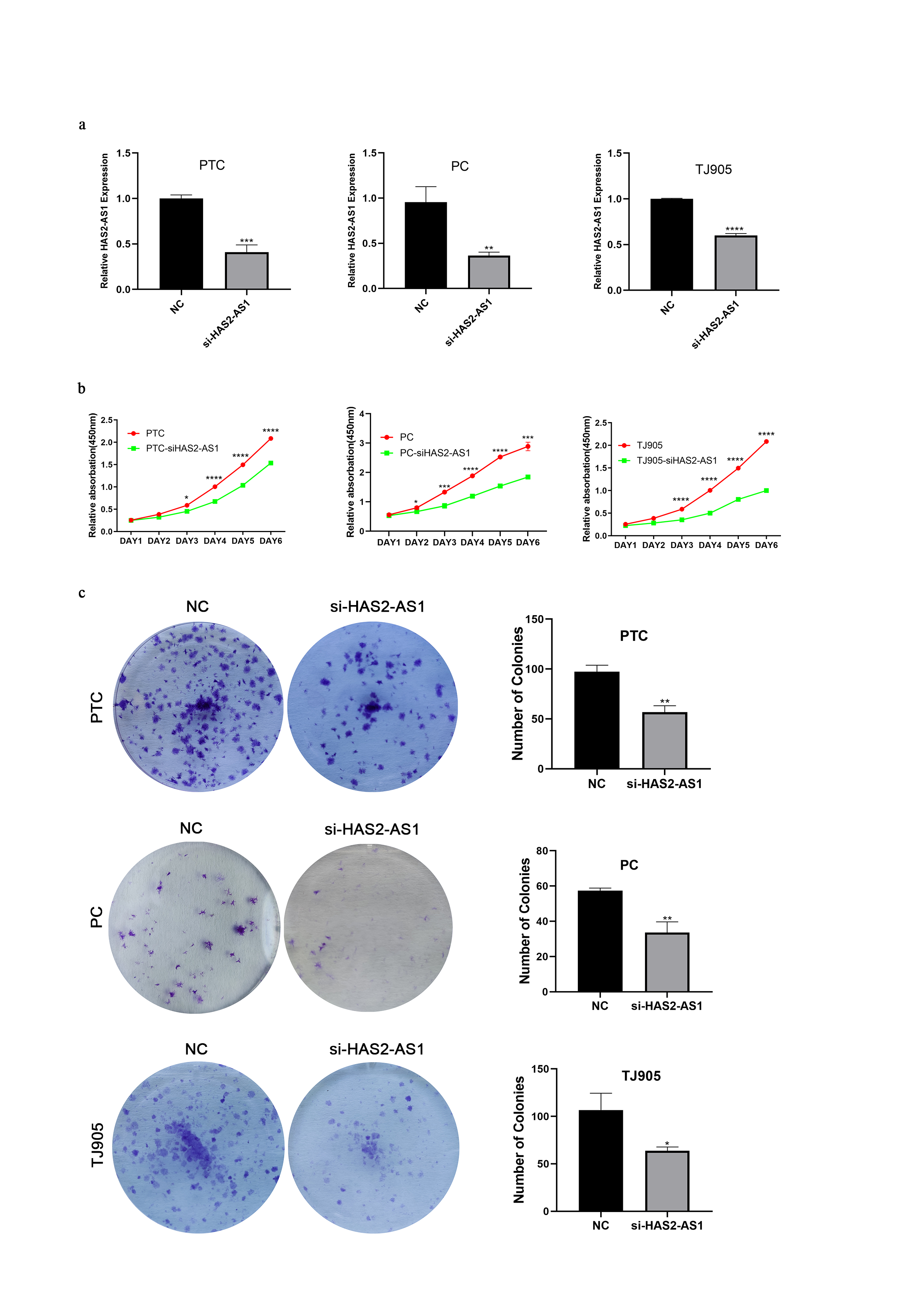

Supplement: Supplementary Figure 1 — (A) HAS2-AS1 expression in PTC, PC and TJ905 cells transfected with siRNA for HAS2-AS1 or scrambled siRNA was measured by qRT-PCR. GAPDH was used as an internal control. (B) CCK-8 assays were conducted to assess the proliferative abilities of PTC, PC and TJ905 cells after HAS2-AS1 knockdown. (C) Colony formation assays were performed to determine the proliferation potential after HAS2-AS1 knockdown in PTC, PC and TJ905 cells. [file Image_1.tif]

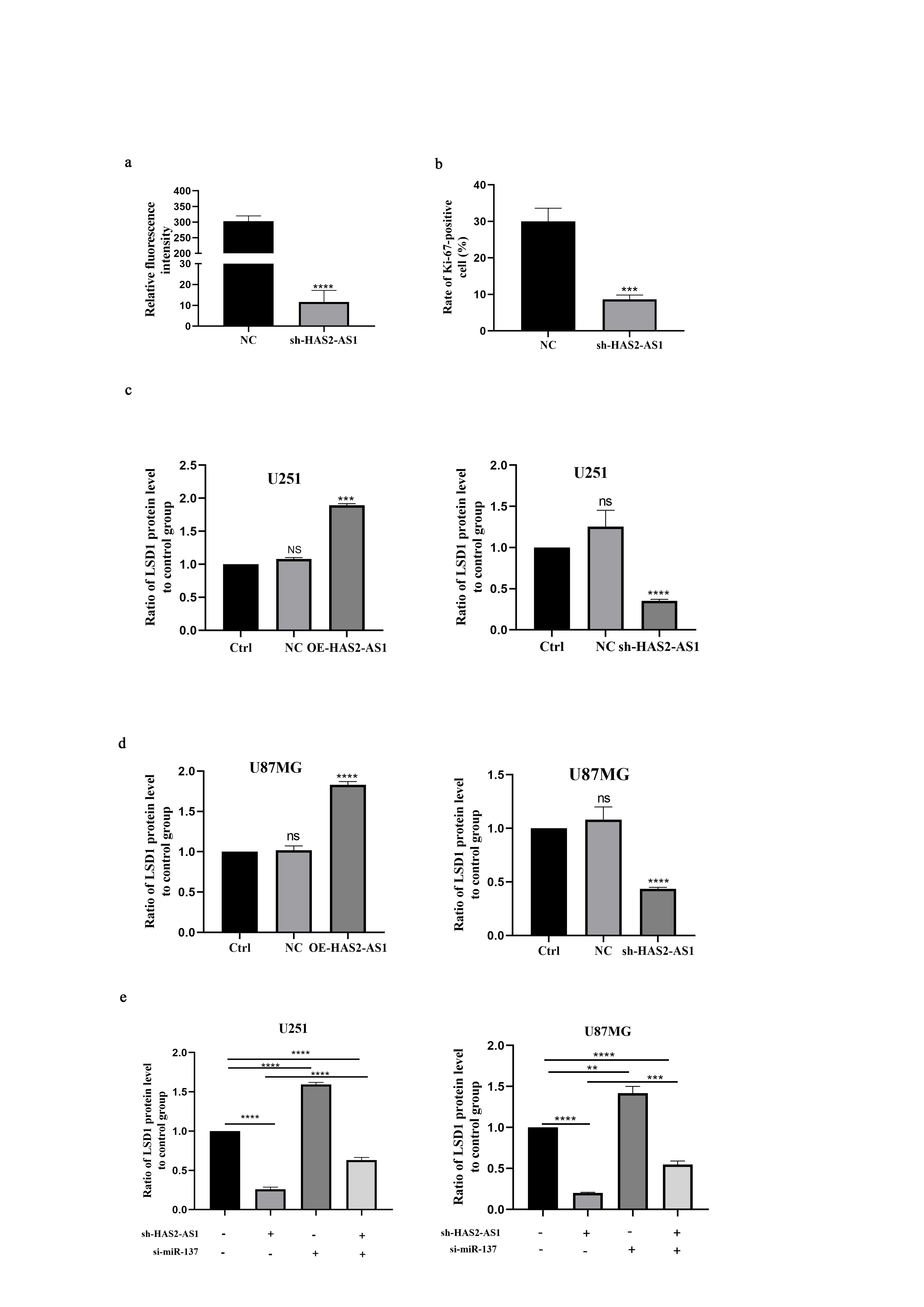

Supplement: Supplementary Figure 2 — (A) Relative fluorescence intensity of Figure 2G. (B) The percentage of Ki-67-positive cells of Figure 2H. (C) Ratio of the LSD1 expression level normalized to that in the control group after overexpression/knockdown of HAS2-AS1 as measured by WB in LNZ308 cells. (D) Ratio of the LSD1 expression level normalized to that in the control group after overexpression/knockdown of HAS2-AS1 as measured by WB in U87 cells. (E) The ratio of the LSD1 expression levels in different groups of LNZ308 and U87 cells normalized to that in the control group. The results showed that the miR-137 inhibitor reversed the change in LSD1 expression caused by HAS2-AS1 silencing at the protein level. [file Image_2.tif]
