# Supplementary material for: LncRNA HAS2-AS1 Promotes Glioblastoma Proliferation by Sponging miR-137
Source: Front Oncol. 2021 May 20;11:634893. doi: 10.3389/fonc.2021.634893 (PMC8173206; doi:10.3389/fonc.2021.634893)
Supplement: Supplementary file 3 [file Table_1.docx]

Table S1 Sequences of lncRNA siRNA, miRNA mimics/inhibitor

| Genes | sense | antisense |
| --- | --- | --- |
| HAS2-AS1 siRNA | 5’-AAGCUGACUUAAAAAGUUAAA-3’ | 5’-UUUAACUUUUUAAGUCAGCUU-3’ |
| scrambled siRNA | 5’-UUCUCCGAACGUGUCACGUdTdT-3’ | 5’-ACGUGACACGUUCGGAGAAdTdT-3’ |
| miR-137 mimics | 5’-UUAUUGCUUAAGAAUACGCGUAG-3’ | 5’-CUACGCGUAUUCUUAAGCAAUAA-3’ |
| Mimics NC | 5’-UCACAACCUCCUAGAAAGAGUAGA-3’ | 5’-UCUACUCUUUCUAGGAGGUUGUGA-3’ |
| miR-137 inhibitor | / | 5’-CUACGCGUAUUCUUAAGCAAUAA-3’ |
| Inhibitor NC | / | 5’-UCUACUCUUUCUAGGAGGUUGUGA-3’ |

Table S2 Sequences of primers for RT-PCR

| Genes | Forward primer | Reverse primer |
| --- | --- | --- |
| HAS2-AS1 | 5’-CGCAAATGGGCGGTAGGCGTG-3’ | 5’-TAGAAGGCACAGTCGAGG-3’ |
| LSD1 | 5’-TTTTGGAAGCCAGGGATCGT-3’ | 5’-GACAGTGTCAGCTTGTCCGTTG-3’ |
| U6 | 5’-CTCGCTTCGGCAGCACA-3’ | 5’- AACGCTTCACGAATTTGCGT-3’ |
| GAPDH | 5’-GGAGCGAGATCCCTCCAAAAT-3’ | 5’-GGCTGTTGTCATACTTCTCATGG-3’ |

Table S3 The RT primers, forward and reverse primers for miR-137/U6.

| Primer | Catalog numbers |
| --- | --- |
| miR-137 RT primer | ssD809230145 |
| U6 RT primer | ssD0904071008 |
| miR-137 forward primer | ssD809230837 |
| miRNA reverse primer | ssD089261711 |
| U6 forward primer | ssD0904071006 |
| U6 reverse primer | ssD0904071007 |

*The miR-137/U6 forward and reverse primers were purchased from Ribobio (Guangzhou, China).
